# Red Blood Cells Beyond Oxygen Transport: Dynamic Regulators, Disease Biomarkers and Therapeutic Platforms—A Narrative Review

**DOI:** 10.3390/ijms27198848

**Published:** 2026-10-03

**Authors:** Joan-Lluis Vives-Corrons

**Affiliations:** 1 Rare Anemias Ektacytometry Unit, Josep Carreras Leukaemia Research Institute, Ctra. de Can Ruti, Camí de les Escoles, 08916 Badalona, Catalonia, Spain; jlvives@somclinic.cat; 2 Medical Centre for Ambulatory Medicine, Carrer de Còrsega 209, 08036 Barcelona, Catalonia, Spain

**Keywords:** red blood cells, erythrocyte deformability, microcirculation, oxidative stress, biomarkers, extracellular vesicles, drug delivery, precision medicine

## Abstract

Red blood cells (RBCs) have traditionally been regarded as passive carriers of respiratory gases. Increasing evidence, however, indicates that they are active participants in vascular homeostasis and respond to mechanical, metabolic, and oxidative stimuli. This narrative review examines the interconnected roles of RBCs as regulators of vascular physiology, indicators of hereditary and systemic disease, therapeutic targets, and delivery platforms. RBC deformability, membrane organization, ion-water balance, metabolism, and redox capacity support microcirculatory flow, while ATP release, nitric oxide handling, adhesion, and extracellular-vesicle formation influence vascular function. RBC morphology, mechanics, hydration, metabolism, oxidative status, and vesiculation can provide clinically relevant information, although emerging functional and molecular biomarkers require further standardization and validation. RBC-directed treatments and RBC-based carriers offer additional therapeutic opportunities. Automated image analysis and machine-learning-assisted microfluidic classification are the most substantiated artificial-intelligence applications; broader multimodal decision-support systems remain exploratory.

## 1. Introduction: Redefining the Red Blood Cell

Red blood cells (RBCs) are the most abundant circulating cells and have traditionally been viewed as highly specialized carriers of oxygen and carbon dioxide. Their structure permits repeated passage through capillaries and the spleen, while their biochemical and mechanical responses influence vascular physiology. Although terminal differentiation eliminates the nucleus and most organelles, mature RBCs remain functionally responsive. Their biconcave geometry and membrane–cytoskeleton organization provide the surface area and flexibility required for repeated capillary and splenic transit [1,2,3,4]. Metabolic regulation and cation-water homeostasis help preserve membrane integrity, cell volume, and deformability during circulation [3,5].

RBCs respond to variations in mechanical stress, oxygen tension, and redox conditions. These inputs can influence cell volume, energy metabolism, antioxidant defense, oxygen unloading, and interactions with the vascular environment [5,6,7,8,9]. The identification of PIEZO1 as a mechanically activated ion channel, together with the clinical consequences of gain-of-function PIEZO1 variants, provides direct molecular evidence that erythrocytes possess clinically relevant mechanosensory pathways [6,10]. This broader perspective places the RBC at the intersection of vascular regulation, disease phenotyping, and therapeutic innovation. Abnormalities of mechanics, hydration, metabolism, redox balance, adhesion, or vesiculation characterize hereditary red-cell disorders and may also reflect systemic diseases. These properties can therefore serve as functional biomarkers and therapeutic targets, while intact RBCs and RBC-derived materials offer biocompatible drug-delivery platforms.

This narrative review examines RBCs as regulators of vascular homeostasis, indicators of hereditary and acquired disease, therapeutic targets, and bioengineered carriers [6,7,8,11].

### Literature Search and Selection

This narrative review was informed by a structured search of PubMed/MEDLINE, Scopus, and Web of Science from database inception through 17 September 2026. Search terms combined “red blood cell” or “erythrocyte” with deformability, mechanotransduction, PIEZO1, ion transport, metabolism, redox biology, extracellular vesicles, biomarkers, ektacytometry, microfluidics, artificial intelligence, drug delivery, and engineered erythroid cells. English-language original studies, consensus recommendations, and authoritative reviews directly relevant to RBC physiology, diagnostics, or therapeutics were considered, and reference lists were screened for seminal publications. Selection emphasized mechanistic relevance, methodological rigor, recency, and clinical importance. Because this was a narrative rather than a systematic review, no formal risk-of-bias assessment or quantitative synthesis was undertaken.

## 2. RBCs as Dynamic Regulators of Vascular Homeostasis

**Figure 1:**
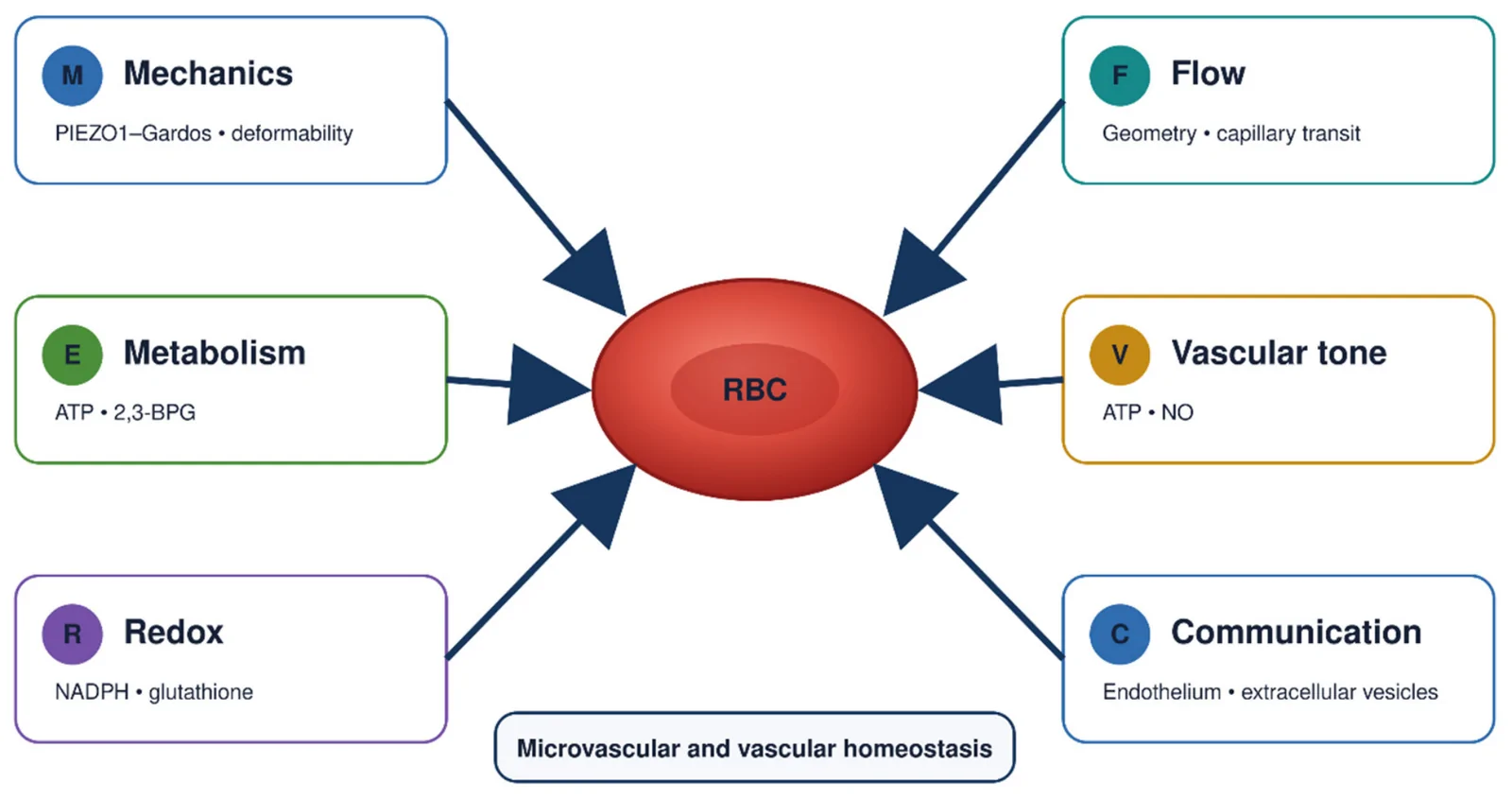
Red blood cells in vascular homeostasis. RBC mechanics, microcirculatory flow, oxygen-dependent metabolism, vascular-tone regulation, redox control, and intercellular communication converge to maintain microvascular perfusion and vascular homeostasis. Arrows indicate the functional relationships between these interconnected regulatory domains. Abbreviations: 2,3-BPG, 2,3-bisphosphoglycerate; ATP, adenosine triphosphate; NADPH, reduced nicotinamide adenine dinucleotide phosphate; NO, nitric oxide; PIEZO1, piezo-type mechanosensitive ion channel component 1; RBC, red blood cell.

Because RBCs continuously encounter changes in vessel diameter, oxygen tension, shear stress, pH, osmolality, and oxidative conditions, they are well positioned to coordinate cellular mechanics with local vascular requirements (Figure 1). Deformability and volume regulation support microcirculatory transit; oxygen-dependent metabolic responses balance ATP production, antioxidant defense, oxygen unloading; and interactions with nitric oxide, ATP, vascular cells, and extracellular vesicles influence vascular tone, inflammation, and coagulation. These interconnected functions, summarized in Table 1, can shift from adaptive to pathogenic when membrane integrity, hydration, metabolism, or redox control is disrupted [5,6,7,9,12,13,14].

### 2.1. Deformability and Microcirculatory Flow

Red blood cells must repeatedly traverse capillaries with internal diameters smaller than their resting diameter and pass through the narrow interendothelial slits of the splenic red pulp. Their ability to deform rapidly and reversibly is therefore essential for maintaining low-resistance microvascular flow and efficient gas exchange. RBC deformability is determined principally by membrane viscoelasticity, cytoplasmic viscosity, and the surface-area-to-volume ratio [3]. These properties are interdependent and are continuously influenced by cell metabolism, intracellular hemoglobin concentration, membrane lipid and protein composition, cytoskeletal integrity, cation-water homeostasis, and the amount of membrane-bound hemoglobin [3,15].

The membrane–cytoskeleton complex provides the mechanical foundation for normal RBC flexibility and stability. The lipid bilayer is anchored vertically to the spectrin-based cytoskeleton through multiprotein complexes centered on band 3–ankyrin and protein 4.1R–actin. Horizontal interactions within the spectrin–actin network distribute mechanical stress across the cell surface, whereas vertical interactions maintain bilayer attachment to the underlying cytoskeleton. Defects in these interactions lead to progressive membrane loss, abnormal cell morphology, and reduced deformability, as observed in hereditary spherocytosis and hereditary elliptocytosis [4].

Cell hydration is an equally important determinant of RBC rheology. Both excessive hydration and dehydration reduce the capacity of the cell to deform, although through different mechanisms. Overhydration decreases the surface-area-to-volume ratio, making the RBC more spherical, whereas dehydration increases intracellular hemoglobin concentration and cytoplasmic viscosity. The discovery of PIEZO1 established a direct molecular link between mechanical forces and volume regulation. Transient activation of PIEZO1 during deformation permits calcium entry, which can activate the calcium-sensitive KCNN4/Gardos channel. The resulting potassium efflux, accompanied by chloride and water loss, restores cell volume after mechanical stress [6]. Excessive or prolonged activation, however, promotes pathological dehydration and impaired deformability, as occurs in PIEZO1-associated hereditary xerocytosis [10].

Mechanical stimulation may also produce short-term changes in RBC rheological behavior. Exposure to physiological levels of shear stress has been reported to improve deformability, suggesting that normal mechanical forces can induce an adaptive response rather than simply causing cumulative damage [16]. By contrast, excessive or prolonged shear stress, oxidative injury, calcium loading, ATP depletion, or membrane–cytoskeleton disruption reduces deformability and may eventually cause hemolysis. Experimental calcium loading decreases RBC deformability through activation of the Gardos channel, whereas nitric oxide donors and pharmacological Gardos-channel inhibition can attenuate this effect [17].

Reduced deformability can increase microvascular resistance, prolong capillary transit, and promote splenic sequestration. Impaired RBC mechanics are clinically relevant in hereditary membrane disorders, sickle cell disease, diabetes, kidney disease, and malaria [4,18,19,20,21]. However, the relationship between in vitro deformability and in vivo perfusion is not linear. Vessel distensibility, endothelial function, plasma viscosity, hematocrit, RBC aggregation, and heterogeneity of the circulating RBC population can partially compensate for, or amplify, an intrinsic mechanical defect. RBC deformability should therefore be interpreted as one component of an integrated microcirculatory system rather than as an isolated measurement [3,22].

### 2.2. Regulation of Vascular Tone

Because RBCs continuously sample the oxygen tension of microcirculation, they are strategically positioned to couple local oxygen demand to vascular perfusion. Several mechanisms have been proposed by which oxygenation and mechanical deformation may be translated into vasoregulatory signals [7].

One proposed pathway involves the release of ATP from RBCs exposed to reduced oxygen tension, acidosis, or mechanical deformation. Extracellular ATP can bind endothelial P2Y receptors, stimulate nitric oxide and prostacyclin production, and generate conducted vasodilator responses that increase blood flow to metabolically active tissues. Pannexin 1 has been identified experimentally as a possible conduit for hypoxia-induced ATP release from human RBCs [23]. Nevertheless, this mechanism remains controversial because small degrees of hemolysis can release sufficient intracellular ATP to confound extracellular measurements. Sikora and colleagues found a close relationship between ATP release and hemolysis under several experimental conditions, indicating that hemolysis must be rigorously quantified before regulated ATP export can be inferred [24]. Thus, RBC-derived ATP may contribute to local perfusion control, but the relative importance of channel-mediated release and hemolysis-associated release under physiological conditions remains unresolved. At present, extracellular ATP is a research measure rather than a validated clinical biomarker of RBC function; interpretation requires simultaneous assessment of hemolysis and stringent control of sample handling.

RBCs are also intimately involved in nitric oxide biology. Deoxygenated hemoglobin can reduce nitrite to nitric oxide and related bioactive nitrogen species, providing a potential mechanism by which falling oxygen saturation promotes hypoxic vasodilation [25]. Additional proposed mechanisms include the formation, transport, and release of S-nitrosothiols and the presence of an RBC-associated nitric oxide synthase. Although the quantitative contribution of each pathway continues to be debated, collectively they support the concept that RBCs participate in the coordination of oxygen delivery and vascular tone.

**Table 1:** Principal mechanisms by which red blood cells regulate vascular homeostasis.

Regulatory Domain	Principal RBC Components or Mechanisms	Physiological Function	Consequences of Dysregulation and Representative References
Deformability	Spectrin-actin cytoskeleton; band 3-ankyrin complex; membrane protein composition and lipids; surface-area-to-volume ratio; membrane-bound hemoglobin	Reversible deformation, capillary transit, and low-resistance microvascular flow	Increased vascular resistance, impaired perfusion, splenic sequestration, and hemolysis [1,2,3,4,8,15]
Mechanotransduction	PIEZO1-mediated Ca^2+^ entry; KCNN4/Gardos-channel activation	Sensing of mechanical forces and regulation of RBC volume	Dehydration, increased cytoplasmic viscosity, and reduced deformability [6,10,17]
Energy metabolism	Glycolysis; ATP production; band 3-enzyme interactions	Maintenance of ion gradients, membrane organization, and cytoskeletal function	ATP depletion, membrane instability, impaired deformability, and premature clearance [5,9]
Oxygen unloading	Rapoport–Luebering shunt; 2,3-BPG; sphingosine-1-phosphate	Regulation of hemoglobin oxygen affinity and adaptation to hypoxia	Inadequate oxygen delivery or maladaptive oxygen unloading [5,9]
Antioxidant defense	Pentose phosphate pathway; NADPH; glutathione; catalase; superoxide dismutase; peroxiredoxin 2	Protection of hemoglobin, membrane lipids, and cytoskeletal proteins	Hemoglobin oxidation, lipid peroxidation, membrane stiffening, and vesiculation [5,8]
Purinergic signaling	ATP release during hypoxia or deformation; endothelial P2Y receptors	Endothelial NO and prostacyclin production; local vasodilation	Impaired matching of perfusion to oxygen demand; interpretation confounded by hemolysis [7,23,24]
Nitrite-NO signaling	Deoxyhemoglobin-mediated nitrite reduction	Potential contribution to hypoxic vasodilation	Disturbed vascular adaptation to hypoxia [25]
NO scavenging	Intracellular hemoglobin; cell-free hemoglobin; hemoglobin-containing microvesicles	Regulation of NO bioavailability	Vasoconstriction, endothelial dysfunction, and platelet activation [12]
Cellular adhesion	RBC interactions with endothelial cells, platelets, and leukocytes	Normally limited; may participate in cellular recognition and clearance	Vascular stasis, endothelial activation, inflammation, and thrombosis [14,18]
Extracellular-vesicle release	Phosphatidylserine-positive vesicles containing lipids, proteins, and hemoglobin	Removal of damaged RBC components and intercellular communication	Thrombin generation, oxidative endothelial injury, NO scavenging, and vasoconstriction [12,13,14,26]

The physiological effects of RBCs depend on the coordinated preservation of membrane mechanics, cellular volume, energy metabolism, redox balance, and hemoglobin compartmentalization. Several protective mechanisms may become pathogenic when RBC integrity is disrupted, or extracellular-vesicle production is excessive. The entries elaborate the six broad aspects in Figure 1. Abbreviations: 2,3-BPG, 2,3-bisphosphoglycerate; ATP, adenosine triphosphate; Ca^2+^, calcium; KCNN4, potassium calcium-activated channel subfamily N member 4; NADPH, reduced nicotinamide adenine dinucleotide phosphate; NO, nitric oxide; PIEZO1, piezo-type mechanosensitive ion channel component 1; RBC, red blood cell.

The relationship between RBCs and nitric oxide is bidirectional. Nitric oxide can improve RBC deformability by modifying cytoskeletal or membrane proteins and limiting calcium-dependent dehydration [17]. Conversely, intact oxygenated RBCs efficiently consume nitric oxide through reactions with hemoglobin. Under physiological conditions, this reaction is restricted by hemoglobin compartmentalization, the unstirred layer surrounding the RBC, and membrane diffusion barriers. Intravascular hemolysis removes these constraints. Cell-free hemoglobin and hemoglobin-containing RBC-derived microvesicles scavenge nitric oxide more rapidly than hemoglobin enclosed within intact cells, decreasing nitric oxide bioavailability and favoring vasoconstriction, platelet activation, and endothelial dysfunction [12]. In sickle cell disease and after hemolysis of transfused RBCs, nitric oxide depletion may amplify vascular dysfunction, although its contribution varies with the cause and magnitude of hemolysis [12].

### 2.3. Metabolic and Redox Regulation

Despite lacking mitochondria, mature RBCs retain a highly active and adaptable metabolic network. Glycolysis supplies the ATP required to maintain membrane phospholipid asymmetry, cytoskeletal organization, ion gradients, cell volume, and deformability. The Rapoport–Luebering shunt generates 2,3-bisphosphoglycerate (2,3-BPG), which decreases hemoglobin oxygen affinity and facilitates oxygen unloading. The pentose phosphate pathway produces NADPH, which is required to maintain glutathione in its reduced form (GSH) and protect hemoglobin and membrane proteins from oxidative damage [5,8].

RBC metabolism is closely linked to hemoglobin oxygenation. When oxygenated, glycolytic enzymes bind to the cytoplasmic domain of band 3, reducing glycolytic flux and directing glucose toward the pentose phosphate pathway. During deoxygenation, hemoglobin competes for band 3 binding and releases these enzymes into the cytoplasm, increasing glycolysis, ATP production, and 2,3-BPG synthesis [5]. This oxygen-dependent metabolic switch allows RBCs to adjust energy production, antioxidant defense, and oxygen release to local conditions.

Sphingosine-1-phosphate provides an additional link between hypoxia, metabolism, and oxygen delivery. Experimental and human high-altitude studies showed that increased RBC sphingosine-1-phosphate promotes glycolysis and 2,3-BPG production, thereby facilitating oxygen unloading during hypoxic adaptation [9]. These observations challenge the concept of the RBC as metabolically static and instead support its role as an oxygen-responsive metabolic sensor.

RBCs are continuously exposed to oxidative stress because of the high intracellular oxygen concentration and spontaneous hemoglobin autoxidation. Their antioxidant defenses include reduced glutathione (GSH), catalase, superoxide dismutase (SOD), peroxiredoxin 2, and the NADPH-generating pentose phosphate pathway. When oxidant production exceeds these protective mechanisms, hemoglobin oxidation, band 3 clustering, lipid peroxidation, phosphatidylserine exposure, membrane stiffening, and vesiculation occur. In patients with retinal vein occlusion, for example, RBC-derived reactive oxygen species and lipid peroxidation were associated with increased membrane viscosity and impaired deformability, supporting a functional connection between RBC redox status and microvascular dysfunction [8]. Oxidative stress may therefore transform RBCs from protective antioxidant reservoirs into amplifiers of vascular injury.

### 2.4. Interactions with Endothelial Cells, Platelets, and Leukocytes

Normal RBCs circulate with minimal adhesion to the vascular wall. In pathological conditions, oxidative membrane damage, phosphatidylserine exposure, altered adhesion molecules, and changes in plasma proteins can promote interactions between RBCs and endothelial cells, platelets, leukocytes, or macrophages. These interactions may slow microvascular flow and contribute to endothelial activation, inflammation, and thrombosis, particularly in sickle cell disease and myeloproliferative neoplasms [14,18].

RBC-derived extracellular vesicles constitute an additional mechanism of vascular communication. Vesiculation removes damaged membrane components and oxidized proteins, helping to preserve the integrity of the parent RBC. The released vesicles, however, retain bioactive lipids, membrane proteins, hemoglobin, and other cytoplasmic constituents. Their biological effects depend on their concentration, cellular origin, molecular composition, and clinical context [12,13,14,26].

Exposure of negatively charged phosphatidylserine provides a catalytic surface for the assembly of coagulation complexes. RBC-derived microparticles isolated from stored blood units can initiate and propagate thrombin generation in plasma [13]. Hemoglobin-containing vesicles may also reduce nitric oxide bioavailability [12], whereas the transfer of oxidant enzymes or other molecular cargo to endothelial cells may modify vascular reactivity. In a JAK2V617F model of myeloproliferative neoplasia, erythrocyte-derived microvesicles were taken up by endothelial cells, increased endothelial oxidative stress, and enhanced arterial vasoconstriction [14].

These findings demonstrate that the contribution of RBCs to vascular homeostasis extends beyond their intracellular functions. Through deformability, oxygen-responsive metabolism, purinergic and nitric oxide signaling, redox regulation, cellular adhesion, and extracellular-vesicle release, RBCs influence microvascular perfusion and endothelial function. The same mechanisms that support physiological adaptation can become pathogenic when membrane stability, hydration, metabolism, or redox control is disturbed [5,6,7,8,12,13,14]. These functions may also leave measurable signatures in circulating RBCs; Section 3 examines their potential and current limitations as disease indicators.

## 3. RBCs as Indicators of Disease

**Figure 2:**
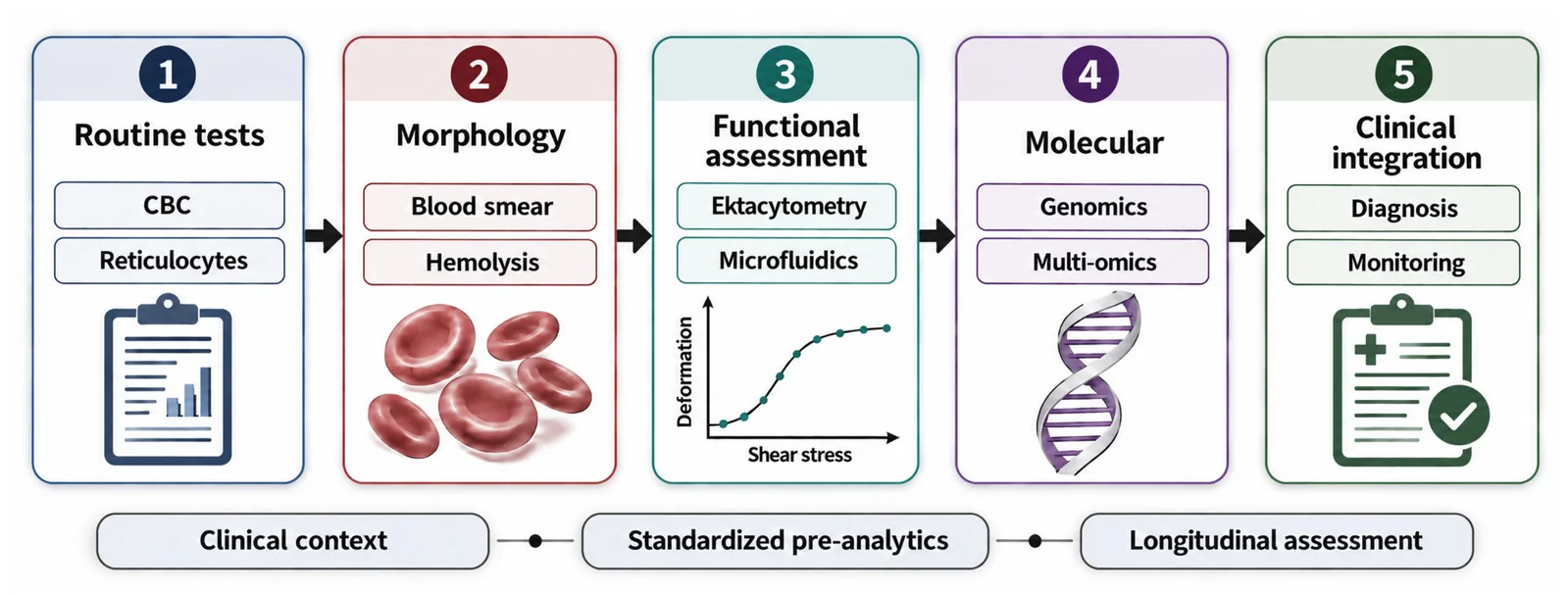
**Integrated red-cell phenotyping.** The numbered panels show the sequential integration of routine testing, morphology, functional assessment, molecular analysis, and clinical interpretation. Blue, red, teal, violet, and green identify these five analytical domains, respectively. The report, RBCs, deformation curve, DNA helix, and checked clinical record represent the principal information generated at each stage. Black arrows indicate information flow, while the lower boxes identify the clinical, pre-analytical, and longitudinal framework required for interpretation. **Abreviation:** CBC, complete blood count.

Throughout their lifespan, RBCs are exposed to changes in oxygen tension, shear stress, plasma composition, inflammatory mediators, metabolic substrates, and oxidative conditions. Consequently, their morphology, mechanical properties, hydration, metabolism, membrane composition, and survival may reflect both primary erythrocyte disorders and systemic disease. Conventional hematological indices provide an initial assessment, whereas newer functional and molecular assays can detect abnormalities that are not evident from hemoglobin concentration or cell morphology alone. This transition from conventional indices to functional and molecular phenotyping is illustrated in Figure 2. RBC-based biomarkers may support diagnosis, disease classification, severity assessment, and monitoring of treatment response. However, many RBC abnormalities are nonspecific and are influenced by reticulocytosis, transfusion, iron status, renal function, inflammation, sample storage, and analytical methods. Interpretation therefore requires integration with the clinical phenotype and other laboratory findings [19,20,22,27,28,29,30,31,32,33]. Representative hereditary RBC disorders are summarized in Table 2.

For clinical interpretation, it is useful to distinguish established biomarkers—including complete blood count indices, reticulocyte count, blood-film morphology, hemolysis markers, hemoglobin analysis, eosin-5-maleimide binding, and disease-specific enzyme assays—from emerging or experimental measures such as oxygen-gradient ektacytometry, single-cell microfluidics, extracellular-vesicle profiling, and omics signatures. Osmotic-gradient ektacytometry (OGE), which is used in specialized diagnostic centers, occupies an intermediate translational position. Emerging measures should not be treated as interchangeable with validated diagnostic tests until analytical standardization and prospective clinical validation are available [27,28,29,30,31,34,35,36,37].

Figure 2 summarizes the complementary levels of RBC phenotyping used to investigate these changes. Routine blood counts, expert morphology and validated disease-specific investigations have established clinical roles. In contrast, proposed microfluidic, molecular, vesicle and integrated AI signatures require indication-specific analytical and clinical validation before routine use.

### 3.1. Conventional Hematological and Morphological Indicators

The complete blood count remains the initial approach to RBC assessment. Hemoglobin concentration and hematocrit identify anemias or erythrocytosis but provide limited information about the underlying mechanism. Mean corpuscular volume (MCV) supports the conventional classification of anemia as microcytic, normocytic, or macrocytic, whereas mean corpuscular hemoglobin (MCH) and mean corpuscular hemoglobin concentration (MCHC) reflect hemoglobin content and cellular concentration. Reticulocyte counts indicate the marrow response and help distinguish hypoproliferative anemia from hemolysis or blood loss.

Automated analyzers provide additional parameters that may reveal abnormalities of RBC volume, hemoglobinization, or hydration. An increased MCHC and an elevated percentage of hyperchromic cells may suggest hereditary spherocytosis or another membrane disorder, although neither finding is specific. Conversely, reduced MCH and MCV are characteristic of iron deficiency and thalassemia but may also occur in chronic inflammation, sideroblastic disorders, or some enzymopathies. Interpretation should therefore consider iron status, reticulocyte count, hemoglobin analysis, and clinical context [4,34].

Red-cell distribution width (RDW) measures heterogeneity of cell volume and is traditionally used to support the differential diagnosis of anemia. Increased RDW may reflect nutritional deficiency, hemolysis, reticulocytosis, ineffective erythropoiesis, transfusion, inflammation, or disturbed erythrocyte maturation. Large population studies have also associated increased RDW with cardiovascular and all-cause mortality, even after adjustment for conventional risk factors [38]. Associations have similarly been reported in heart failure, sepsis, kidney disease, and cancer. Nevertheless, RDW is a nonspecific risk marker rather than a direct measure of a single biological pathway, and its prognostic associations may reflect the combined effects of inflammation, oxidative stress, impaired erythropoiesis, nutritional deficiency, and reduced RBC survival. Reverse causation and residual confounding are possible: underlying illness can raise RDW while independently increasing mortality, so observational associations do not establish that RDW itself causes adverse outcomes.

Peripheral blood morphology remains indispensable because it can reveal abnormalities that are averaged or missed by automated indices. Spherocytes, elliptocytes, stomatocytes, target cells, schistocytes, sickle cells, bite cells, echinocytes, acanthocytes, Howell–Jolly bodies, Heinz bodies, and basophilic stippling provide diagnostic clues to membrane defects, hemoglobinopathies, oxidative injury, mechanical fragmentation, hyposplenism, and dyserythropoiesis. However, morphological interpretation is partly observer-dependent and is influenced by smear preparation, staining, storage, and the proportion of abnormal cells. Digital imaging and artificial intelligence may improve reproducibility, but expert morphological assessment remains important, particularly in rare or mixed disorders [34,39].

### 3.2. Functional and Biomechanical Biomarkers

RBC deformability integrates membrane elasticity, surface-area-to-volume ratio, intracellular viscosity, cell hydration, and metabolic status. It can be assessed by filtration, micropipette aspiration, optical tweezers, conventional shear-stress ektacytometry, osmotic gradient ektacytometry, oxygen gradient ektacytometry, and microfluidic devices. Each method measures a related but not identical aspect of cell mechanics; consequently, results obtained using different platforms are not necessarily interchangeable [3,22,27,29].

Circulating RBC populations are intrinsically heterogeneous because cells span the reticulocyte-to-senescent-cell continuum and differ in age, reticulocyte content, density, hydration, metabolic and redox state, membrane loss, and accumulated oxidative or mechanical damage [1,2]. Consequently, bulk or population-average measurements may obscure small but clinically relevant subpopulations with distinct deformability, microvascular transit, or adhesive behavior. Single-cell microfluidic and imaging methods can characterize these distributions and identify outlier populations, but their analytical and diagnostic performance depends on the protocol, clinical condition, and reference standard; recent transfusion and altered erythropoiesis may further complicate interpretation. These methods require standardized sampling, processing, analysis, and reporting [22,39,40].

Osmotic gradient ektacytometry (OGE) evaluates RBC deformability across a continuous osmotic gradient and generates a characteristic curve reflecting cell geometry, membrane surface area, hydration, and intracellular viscosity. The principal parameters include Omin, which is related to the osmotic fragility of the cells; EImax, representing maximal deformability; and Ohyper, which is influenced by cellular hydration and cytoplasmic viscosity. Distinctive curve profiles may support the differential diagnosis of hereditary spherocytosis, hereditary elliptocytosis, hereditary pyropoikilocytosis, hereditary xerocytosis, and overhydrated hereditary stomatocytosis [27]. However, recent transfusion can produce mixed or near-normal curves, and some conditions may generate overlapping profiles. For example, a 2026 single-laboratory cohort of 181 samples referred for suspected hereditary spherocytosis reported 96.0% sensitivity and 96.9% specificity for EMA binding at its study-specific optimal threshold; these estimates should not be extrapolated to other disorders or protocols [35].

Combining functional testing with molecular analysis can considerably improve diagnostic interpretation. In a series of hereditary membranopathies, the combination of targeted next-generation sequencing (t-NGS) and OGE linked genotype with membrane behavior and helped clarify cases with inconclusive morphology or conventional laboratory findings [28]. This approach is particularly valuable when a variant of uncertain significance is detected, because a concordant functional phenotype provides evidence that the molecular alteration affects RBC physiology.

Oxygen gradient ektacytometry, measured with the oxygen module, evaluates the dynamic response of sickle RBCs during controlled deoxygenation and reoxygenation. It measures normoxic deformability, minimum deformability during hypoxia, the oxygen tension at which sickling begins, and recovery after reoxygenation. These parameters incorporate the effects of hemoglobin S concentration, fetal hemoglobin, RBC hydration, membrane mechanics, and treatment [29]. Some studies have associated oxygen-gradient parameters with vaso-occlusive complications and therapeutic response, but other studies have not demonstrated independent prediction of pain episodes [30,31]. The method is therefore promising for functional characterization and treatment monitoring, but its prognostic role requires further prospective validation.

Microfluidic systems more closely reproduce capillary dimensions and can evaluate single-cell transit, occlusion, adhesion, deformability, and recovery under controlled flow and oxygen conditions. Measurements based on the pressure required for RBC passage through microconstrictions can detect small and reproducible differences in cortical tension [22]. Microfluidic platforms may be especially useful when disease heterogeneity is clinically relevant, because population-average measurements can obscure small subpopulations of highly rigid or adhesive cells. Their translation to routine practice will depend on analytical standardization, throughput, cost, and demonstration of added clinical value.

### 3.3. Hereditary RBC Disorders

In hereditary RBC disorders, the erythrocyte is both the principal site of pathology and a direct indicator of the underlying molecular defect. Membranopathies alter membrane stability, cell geometry, ion permeability, or hydration. Hereditary spherocytosis is typically associated with reduced surface area, increased osmotic fragility, decreased EImax, and a leftward shift of Omin. Hereditary elliptocytosis and hereditary pyropoikilocytosis predominantly affect membrane mechanical stability and produce characteristic morphological and ektacytometric abnormalities. PIEZO1- or KCNN4-associated hereditary xerocytosis causes cellular dehydration, increased intracellular viscosity, and a rightward displacement of the hypertonic region of the osmoscan curve [4,27,28].

Hemoglobinopathies generate different functional signatures. In sickle cell disease (SCD), hemoglobin S polymerization during deoxygenation causes progressive cell stiffening, sickling, delayed capillary transit, adhesion, and vascular occlusion. Oxygen gradient ektacytometry captures these dynamic changes and can demonstrate the effects of genotype, fetal hemoglobin, transfusion, and disease-modifying treatment [29,30,31]. Thalassemia is associated with microcytosis, membrane oxidative injury, ineffective erythropoiesis, and a heterogeneous population of circulating RBCs with altered deformability and hydration.

Enzymopathies primarily affect energy metabolism or antioxidant defense. Pyruvate kinase deficiency (PKD) reduces ATP generation, compromising membrane homeostasis and RBC survival, whereas glucose-6-phosphate dehydrogenase (G6PD) deficiency impairs NADPH generation and increases susceptibility to oxidant-induced hemoglobin denaturation and membrane damage. Because biochemical enzyme activity may be affected by reticulocytosis or transfusion, functional findings should be integrated with molecular analysis [36,37].

Congenital dyserythropoietic anemias (CDAs) are inherited disorders of erythroid maturation characterized by ineffective erythropoiesis and distinctive bone-marrow abnormalities. Circulating findings may include macrocytosis or anisopoikilocytosis, an inadequate reticulocyte response, variable hemolysis, and secondary iron overload. Because morphological findings overlap among CDA subtypes and with other inherited anemias, molecular confirmation is often required [41]

Hereditary anemias encompass diverse defects of the membrane, metabolism, hemoglobin, and erythroid maturation; consequently, no single RBC assay is sufficient for every disorder. The most informative strategy combines family history, blood count, morphology, hemolysis markers, disease-specific biochemical tests, deformability or hydration analysis when appropriate, and genomic findings [28,36,37,41]. Table 2 summarizes the dominant RBC phenotypes, informative assessments, and principal interpretation caveats for selected hereditary disorders.

**Table 2:** Representative red blood cell alterations in hereditary RBC disorders.

Disorder Group	Dominant RBC Phenotype	Most Informative Assessments	Main Interpretation Caveat and Representative References
Membranopathies: HS; HE/HPP	Surface-area loss, spherocytes or elliptocytes, membrane instability, reduced deformability, and abnormal osmotic-gradient profiles	Blood film; EMA binding; osmotic-gradient ektacytometry; membrane-protein analysis; targeted NGS	Mild phenotypes and recent transfusion can mask characteristic findings [4,27,28,35]
**Cation-leak disorders: hereditary xerocytosis**	RBC dehydration, increased MCHC, reduced osmotic fragility, and right-shifted hypertonic osmoscan region	Osmoscan; PIEZO1/KCNN4 sequencing	Transfusion may normalize the curve; splenectomy requires caution [6,10,27,28]
**Hemoglobinopathies: SCD; thalassemia**	Hypoxia-induced sickling, dense cells, microcytosis, target cells, oxidative injury, and heterogeneous deformability	Hemoglobin analysis; oxygen-gradient ektacytometry; microfluidics; HbF; morphology	Results are influenced by genotype, HbF, iron status, transfusion, and treatment [29,30,31]
**Enzymopathies: PK deficiency; G6PD deficiency**	ATP depletion or impaired antioxidant defense, hemolysis, membrane instability, and oxidant-induced Heinz bodies or bite cells	Enzyme activity; redox or metabolomic assays; PKLR or G6PD sequencing	Reticulocytosis and transfusion can distort enzyme activity [36,37]
Dyserythropoietic disorders: congenital dyserythropoietic anemia (CDA)	Ineffective erythropoiesis, macrocytosis or anisopoikilocytosis, erythroid dysplasia, hemolysis, and secondary iron overload	CBC and blood film; hemolysis and iron studies; bone-marrow morphology; targeted NGS	Morphology overlaps across subtypes; molecular confirmation is often required [41]

This condensed table groups related hereditary RBC disorders by dominant pathophysiological pattern to improve readability. Individual diagnoses still require integration of morphology, functional testing, biochemical assays, molecular findings, transfusion history, reticulocyte count, iron status, splenectomy status, and clinical context. The grouped labels do not imply equal severity: HS and HE, in particular, have broad and distinct clinical spectra. EMA binding in one single-center cohort of 181 suspected HS cases showed 96.0% sensitivity and 96.9% specificity at a study-specific cut-off [35]; these values are not transferable to the grouped disorders. Disease-specific test performance should be assessed in the appropriate population. Abbreviations: ATP, adenosine triphosphate; CBC, complete blood count; EMA, eosin-5-maleimide; G6PD, glucose-6-phosphate dehydrogenase; HbF, fetal hemoglobin; HE/HPP, hereditary elliptocytosis/hereditary pyropoikilocytosis; HS, hereditary spherocytosis; MCHC, mean corpuscular hemoglobin concentration; NGS, next-generation sequencing; PK, pyruvate kinase; RBC, red blood cell; SCD, sickle cell disease.

### 3.4. RBC Alterations in Acquired and Systemic Disease

RBC abnormalities also occur when the erythrocyte is not the primary disease target. Hyperglycemia, oxidative stress, nonenzymatic glycation, altered plasma lipids, and low-grade inflammation impair membrane fluidity and deformability in diabetes. Reduced deformability has been detected before advanced nephropathy and becomes more pronounced as renal function deteriorates [19]. These changes may contribute to microvascular resistance but also act as indicators of cumulative metabolic and vascular injury.

Chronic kidney disease affects RBC production, lifespan, metabolism, and mechanical behavior. In addition to erythropoietin deficiency and disturbed iron metabolism, uremic toxins, oxidative stress, inflammation, and membrane lipid alterations shorten RBC survival. Experimental and clinical investigations have linked proteinuric kidney disease with reduced RBC lifespan, altered deformability, phosphatidylserine exposure, and metabolic reprogramming [20]. RBC analysis may therefore provide information complementary to hemoglobin concentration, although the effects of iron therapy, erythropoiesis-stimulating agents, dialysis, and transfusion must be considered.

In sepsis, increased RDW has been associated with mortality in elderly patients [32]. This association does not establish a specific erythrocyte mechanism, and RDW should be interpreted as a nonspecific risk marker that may integrate inflammation, altered erythropoiesis, nutritional status, and changes in RBC survival. Functional RBC measurements may help characterize microcirculatory dysfunction, but clinically useful thresholds and prospective validation are still required.

In cancer, tumor-related inflammation, nutritional deficiency, renal dysfunction, treatment toxicity, and altered erythropoiesis may affect conventional RBC indices. Proteomic studies have also identified disease-associated changes in RBC proteins; for example, distinct RBC protein profiles have been reported in patients with breast cancer and at different tumor stages [33]. However, these findings remain exploratory and require independent validation before clinical use.

In malaria, parasite invasion profoundly remodels the host RBC by altering membrane proteins, cytoskeletal organization, rigidity, adhesion, and permeability. Reduced deformability of infected and, to a lesser extent, uninfected circulating RBCs contributes to splenic clearance and microvascular obstruction [21]. Malaria therefore illustrates how RBC properties can record cumulative cellular exposure during circulation, although the broader use of RBCs as longitudinal “circulating biosensors” requires disease-specific clinical validation.

### 3.5. Emerging Molecular and Multiparametric Indicators

Genomic findings do not always establish the functional relevance of a detected variant. In hereditary anemias, concordant abnormalities in morphology, deformability, hydration, ion transport, or enzyme activity can help determine whether a variant of uncertain significance alters RBC physiology [28].

Proteomic, metabolomic, lipidomic, and ion-transport analyses provide complementary molecular descriptions of membrane organization, oxidative damage, energy metabolism, lipid remodeling, and cation homeostasis. Because no single study covers all these layers, this broader concept is supported by complementary evidence from oxygen-dependent metabolome analysis, hypoxic metabolic adaptation, disease-associated RBC metabolism, and RBC proteomics [5,9,20,42]. RBC-derived extracellular vesicles may add information about cellular aging, membrane stress, hemolysis, inflammation, and coagulation, but their measurement is highly sensitive to collection, processing, storage, and analytical methodology [26].

Digital morphology, ektacytometry, microfluidics, and molecular profiling therefore characterize different levels of the RBC phenotype. Their value lies in complementarity: conventional indices establish the hematological context, morphology reveals cellular heterogeneity, functional assays quantify physiological impairment, and molecular analyses identify the pathways responsible [22,27,28,29,30,31,39,42].

## 4. RBCs as Therapeutic Targets

**Figure 3:**
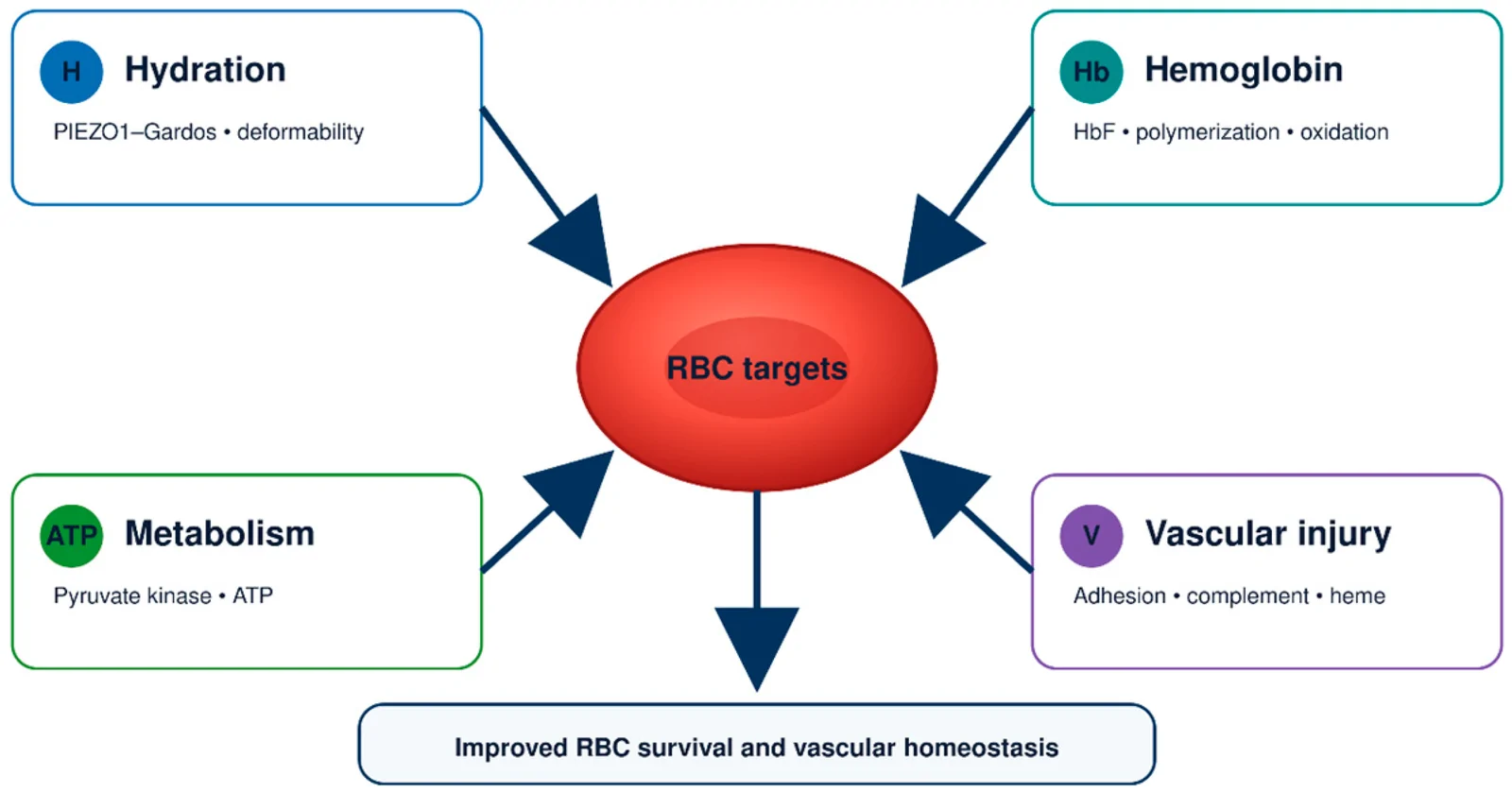
**Red blood cells as therapeutic targets.** Principal strategies act on membrane hydration and deformability, hemoglobin function, metabolism and survival, or pathological vascular interactions. Inward arrows indicate interventions directed toward RBC targets, whereas the downward arrow represents their shared objective of improving RBC survival and vascular homeostasis. **Abbreviations:** ATP, adenosine triphosphate; Hb, hemoglobin; HbF, fetal hemoglobin; PIEZO1, piezo-type mechanosensitive ion channel component 1; RBC, red blood cell.

The molecular mechanisms governing RBC deformability, hydration, hemoglobin function, metabolism, and vascular interactions provide multiple therapeutic targets. Rather than correcting anemia alone, current strategies increasingly aim to restore the functional properties of circulating RBCs, prolong their survival, and reduce the vascular consequences of hemolysis [43,44,45,46,47,48,49]. The principal therapeutic domains are compared in Table 3 and illustrated in Figure 3.

### 4.1. Correcting Membrane and Hydration Abnormalities

Pathological dehydration increases intracellular hemoglobin concentration, cytoplasmic viscosity, and membrane rigidity. This is particularly relevant in PIEZO1- and KCNN4-associated hereditary xerocytosis and in sickle cell disease. PIEZO1 activation permits Ca^2+^ entry, which stimulates the KCNN4/Gardos channel and leads to potassium and water loss. Pharmacological modulation of this pathway may therefore prevent dehydration and improve deformability. Gardos-channel inhibition reduces dense-cell formation and hemolysis; however, a phase III senicapoc trial was stopped early for lack of efficacy and failed to reduce painful crises in sickle cell disease, demonstrating that rheological improvement does not necessarily translate into clinical benefit [6,10,43].

Direct PIEZO1 modulation remains mainly experimental because the channel has important functions in several tissues. Alternative approaches include selective inhibition of downstream pathways and stabilization of membrane–cytoskeleton interactions. Antioxidant or metabolic interventions may additionally limit band 3 oxidation, lipid peroxidation, phosphatidylserine exposure, and vesiculation. Their efficacy should be assessed using functional measurements of hydration, deformability, and RBC survival rather than biochemical markers alone [6,8,10].

### 4.2. Modifying Hemoglobin Function

Inducing fetal hemoglobin remains one of the most effective treatments for sickle cell disease. Hydroxyurea increases fetal hemoglobin and reduces painful crises in a randomized clinical trial [44]. Experimental work in humanized sickle cell mice also found increased nitric oxide bioavailability after hydroxyurea treatment [45], although the contribution of this mechanism to clinical benefit remains uncertain. More recently, gene-editing strategies targeting the erythroid enhancer of BCL11A have shown that sustained fetal hemoglobin reactivation can substantially reduce severe vaso-occlusive events [46]. This autologous approach nevertheless requires stem-cell collection and myeloablative conditioning, with associated toxicity, specialist infrastructure, and substantial cost. Long-term monitoring is also required to assess late effects and potential unintended genome-editing events [46].

Hemoglobin oxygen affinity has been modified pharmacologically to delay deoxygenation-dependent hemoglobin S polymerization. Voxelotor provided clinical proof of this mechanism, but the associated increase in oxygen affinity may offset gains in oxygen delivery to tissues [50]. In September 2024, the manufacturer withdrew voxelotor worldwide, including from the United States; both the European Medicines Agency and the US Food and Drug Administration issued safety communications after emerging concerns about vaso-occlusive complications and deaths [51,52]. Consequently, oxygen-affinity modulation remains a mechanistically informative but currently unestablished therapeutic strategy; direct polymerization inhibitors and approaches that reduce RBC dehydration continue to be investigated.

Oxidized or unstable hemoglobin damages membrane proteins and lipids, promotes band 3 clustering, and accelerates clearance. Interventions that preserve hemoglobin in its functional reduced state or limit heme-mediated oxidative injury may therefore improve both RBC survival and vascular homeostasis [8,49].

### 4.3. Improving RBC Metabolism and Survival

Pyruvate kinase (PK) activation provides clinical proof that RBC metabolism is therapeutically modifiable. Mitapivat, an oral allosteric activator of PK, increases glycolytic activity and ATP production. In adults with pyruvate kinase deficiency (PKD) who were not regularly transfused, mitapivat increased hemoglobin, reduced hemolysis, and improved patient-reported outcomes [47]. Restoration of ATP supports membrane–cytoskeleton interactions, cation pumps, phospholipid asymmetry, and deformability.

Enhancement of antioxidant defense remains potential rather than an established therapeutic strategy. Increasing NADPH or reduced glutathione availability could protect hemoglobin and membrane components from oxidative injury [8]. Outside PKD, however, clinical evidence that metabolic manipulation improves congenital anemias remains limited; effects on iron metabolism, oxygen affinity, and erythroid maturation require disease-specific evaluation.

### 4.4. Targeting Pathological RBC Interactions

Abnormal RBC adhesion to endothelial cells contributes to microvascular obstruction, particularly in sickle cell disease. Therapeutic strategies include inhibition of adhesion molecules, endothelial activation, inflammatory signaling, and platelet–leukocyte interactions. Because vaso-occlusion is multifactorial, blocking an individual adhesion pathway may be insufficient in all patients [18].

Complement inhibition has transformed the treatment of paroxysmal nocturnal hemoglobinuria (PNH) by reducing intravascular hemolysis and its thrombotic consequences [48]. C3 inhibition with pegcetacoplan has also demonstrated clinical benefit in patients with persistent anemia during C5 inhibition, including patients previously treated with eculizumab [53]. Proximal complement inhibition requires careful infection-risk management and ongoing monitoring.

Finally, hemolysis releases cell-free hemoglobin, heme, and iron, which deplete nitric oxide, promote oxidative stress and inflammation, and contribute to endothelial dysfunction. Hemoglobin- and hem scavenging proteins, particularly haptoglobin and hemopexin, therefore represent promising therapeutic approaches, although their broader clinical use remains under investigation [49]. Together, these strategies show that RBC-directed therapies may improve not only anemia but also microvascular perfusion and systemic vascular homeostasis.

## 5. RBCs as Therapeutic Carriers and Bioengineered Platforms

**Figure 4:**
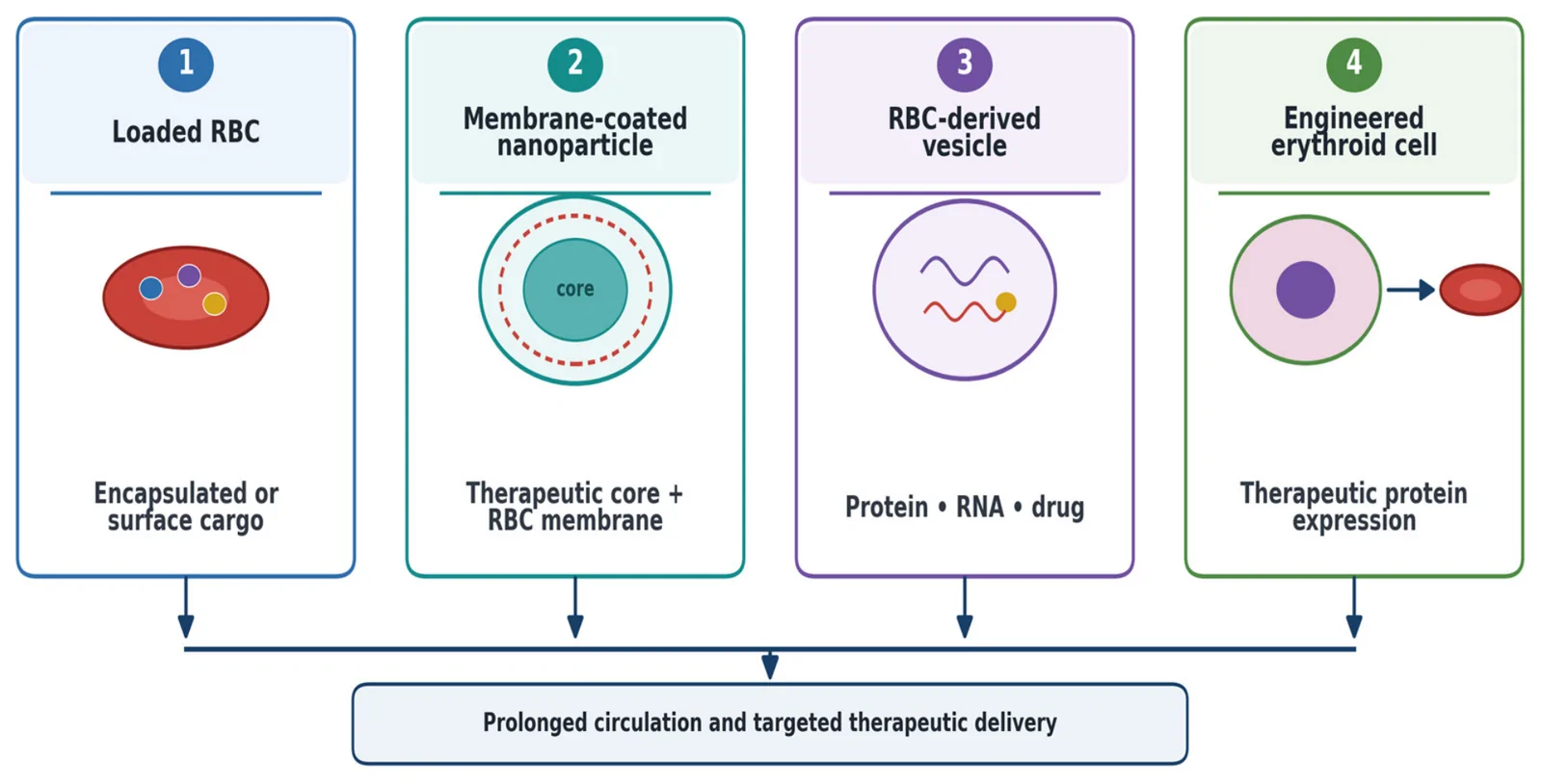
Red-cell-based therapeutic carriers and bioengineered platforms. Four carrier strategies are shown: blue, intact RBCs loaded intracellularly or modified at the surface; teal, RBC-membrane-coated nanoparticles; violet, RBC-derived extracellular vesicles; and green, engineered erythroid cells. Within the nanoparticle symbol, the teal center represents the therapeutic core and the red dashed ring represents the RBC-membrane coating. Solid navy arrows converge on the shared goal of prolonged circulation and targeted therapeutic delivery. Abbreviations: RBC, red blood cell; RNA, ribonucleic acid.

The long circulatory lifespan, biocompatibility, deformability, and large intracellular volume of RBCs make them attractive carriers for therapeutic agents (Figure 4).

Their membrane can also camouflage synthetic nanoparticles, while RBC-derived extracellular vesicles and engineered erythroid precursors provide additional delivery platforms. These approaches seek to prolong drug exposure, reduce immunogenicity, and achieve tissue-selective delivery. However, manipulation must preserve membrane integrity, deformability, hemocompatibility, and predictable clearance [11,54,55,56,57,58,59,60]. The principal RBC-based carrier systems, their construction, and their development status are compared in Table 4.

### 5.1. RBC-Based Drug Delivery

Therapeutic agents can be encapsulated within RBCs or attached to their external membrane. Encapsulation, frequently achieved by controlled hypotonic dialysis and membrane resealing, has been investigated for enzymes, small molecules, corticosteroids, and biological agents [11,54]. Intracellular loading protects susceptible cargo from plasma degradation and immune recognition and may provide sustained release or delivery to macrophage-rich organs. Erythrocyte-encapsulated thymidine phosphorylase has been administered to three patients with mitochondrial neurogastrointestinal encephalomyopathy in an early single-center study; this provides evidence of human use, not regulatory approval [61]. Surface attachment allows therapeutic proteins, antibodies, and nanoparticles to remain accessible to circulating substrates or vascular targets. Nevertheless, excessive surface loading may alter membrane curvature, aggregation, complement activation, or microvascular transit. Autologous RBCs minimize alloimmune incompatibility but require individualized collection and manufacturing. Donor-derived platforms may facilitate standardized production, although they introduce requirements for blood-group compatibility, infectious-disease screening, and control of storage-related alterations [11,54].

### 5.2. RBC Membrane-Coated Nanoparticles

RBC membrane-coated nanoparticles comprise a synthetic core enclosed within a biologically derived membrane shell. The core enables drug loading, controlled release, or imaging, whereas the membrane supplies native proteins and “self” signals that reduce phagocytic recognition and prolong circulation [55]. Targeting ligands can be incorporated to enhance tissue selectivity. These biomimetic platforms are being investigated for cancer, infectious and inflammatory diseases, toxin neutralization, and vascular delivery.

RBC hitchhiking is a distinct approach in which nanoparticles attach transiently to intact RBCs; it increased delivery to selected organs in mice and pigs, with additional testing in ex vivo human lungs [56]. Neither human therapeutic efficacy nor efficacy in non-human primates has been established. Translation requires control of membrane coating and protein orientation for coated particles, attachment stability and release for hitchhiking, and scale-up, sterility and safety for both platforms.

### 5.3. RBC-Derived Extracellular Vesicles

RBC-derived extracellular vesicles are membrane-enclosed particles that can transport proteins, lipids, RNA, and therapeutic compounds. Their characterization requires standardized reporting of collection, processing, and analytical methods [26]. Experimental production and cargo-loading strategies are beginning to permit more reproducible generation of vesicle-based delivery systems with defined properties [57].

Experimental platforms have delivered small interfering RNA, chemotherapeutic agents, and immunomodulatory molecules. In an acute myeloid leukemia model, targeted RBC-derived vesicles carrying doxorubicin and silDO1 enhanced chemo-immunotherapeutic activity [58]. However, phosphatidylserine exposure, residual hemoglobin, batch variability, and methodological heterogeneity may affect coagulation, oxidative stress, clearance, and measured potency; standardized characterization and safety testing are therefore essential [26,58].

### 5.4. Engineered Erythroid Cells

Ex vivo modification of erythroid progenitors provides an alternative to manipulating mature RBCs. Precursors can be genetically engineered to express therapeutic proteins, membrane-bound ligands, or intracellular cargo before enucleation. The resulting cells may combine prolonged circulation with reduced risks associated with persistent nuclear genetic material.

Engineered RBCs expressing immunostimulatory proteins have demonstrated antitumor activity in preclinical models [59]. Immortalized erythroid progenitor lines may eventually support standardized cellular products [60]. Translation will require efficient expansion and enucleation, physiological hemoglobinization, preserved deformability, genetic stability, scalable manufacturing, and rigorous regulatory characterization.

## 6. Integration into Precision Medicine

Precision red-cell medicine should convert the complementary measurements described above into clinically interpretable profiles. Rather than repeating individual assays, the objective is to identify combinations that explain phenotypic variability, detect clinically important subpopulations, and add information beyond routine hematology [22,27,28,29,30,31,42]. A practical pathway should begin with the blood count, blood film, and hemolysis tests and add disease-directed assays only when they address a defined diagnostic question.

The composition of each profile should be determined by the clinical question. Diagnostic evaluation may combine molecular findings with morphology, osmotic-gradient ektacytometry, intracellular cations, or enzyme activity. Patient stratification may incorporate oxygen-gradient ektacytometry, single-cell microfluidics, and molecular signatures to distinguish differences in sickling, hydration, or deformability among patients with the same diagnosis [22,27,28,29,30,31,37].

Longitudinal monitoring should use mechanism-specific endpoints: hydration and deformability for ion-channel modulators, ATP and hemolysis for metabolic therapy, hypoxic deformability for antisickling treatment, and adhesion or vesiculation for vascular interventions. This approach may reveal a biological response before it is apparent from hemoglobin concentration or clinical events.

Artificial-intelligence applications should be distinguished by their level of maturity. Automated RBC image segmentation and classification and machine-learning-assisted analysis of microfluidic phenotypes have been demonstrated in curated research datasets [39,40]. By contrast, integration of ektacytometric curves, microfluidic measurements, multi-omics, and longitudinal clinical outcomes into decision-support models remains exploratory and has not undergone prospective multicenter clinical validation.

As summarized in Table 5, adoption will require harmonized preanalytical procedures, calibrated instruments, appropriate reference ranges, interoperable data formats, interpretable models, and prospective multicenter validation. The domains in Table 5 are ordered by practical readiness: routine morphology and established diagnostic tests precede specialized functional studies, whereas omics and cellular-communication assays remain largely research tools.

Clinical adoption also depends on cost-effectiveness. Instruments, reagents, specialist staff, quality control, throughput and turnaround time should be evaluated against the diagnostic yield and clinical decisions added to routine testing. A tiered assessment, reserving advanced assays for unresolved or high-impact cases, may be more feasible than applying all domains to every patient; its value requires prospective health-economic evaluation.

## 7. Challenges and Future Directions

The translation of RBC research into routine clinical practice is limited by incomplete standardization of functional assays. Ektacytometry, microfluidic deformability testing, and extracellular-vesicle analysis may differ in instrumentation, experimental conditions, data processing, and reported parameters, reducing comparability between laboratories [22,26,62]. Preanalytical variables are equally important: storage duration, temperature, transportation, anticoagulant choice, and repeated temperature changes can alter RBC morphology, metabolism, hydration, and deformability [26,63]. Harmonized collection protocols, quality-control materials, reporting standards, and method-specific reference intervals are therefore essential.

Biological and clinical heterogeneity further complicates interpretation. Reticulocytosis, recent transfusion, inflammation, renal dysfunction, and treatment can modify RBC measurements independently of the primary disorder. These variables should be recorded prospectively and incorporated into study design and analysis. Longitudinal measurements before and after disease progression or therapeutic intervention are required to distinguish potentially causal RBC abnormalities from secondary or nonspecific changes; cross-sectional associations alone cannot establish directionality [26,62,63].

RBC-based therapies present additional translational challenges. Encapsulated drugs, membrane-coated nanoparticles, extracellular vesicles, and engineered erythroid cells require reproducible manufacturing, standardized potency assays, sterility, batch consistency, and preservation of membrane integrity and deformability. Potential complement activation, phosphatidylserine exposure, thrombogenicity, altered biodistribution, immunogenicity, and premature clearance must also be evaluated during long-term safety studies [11,26,54,55,56,57,58,59,60].

Emerging experimental systems may help overcome some of these limitations. Microfluidic platforms can reproduce microvascular constrictions and aspects of splenic filtration under controlled conditions while enabling analysis and classification of heterogeneous RBC populations [40]. Patient-specific microvascular modeling in radiotherapy provides an analogy for the digital-twin concept rather than direct evidence in RBC disorders [64]. Comparable RBC-focused models could eventually incorporate rheology, oxygen transport, and treatment effects, but their application to red-cell disease remains conceptual.

The ultimate objective is to develop clinically applicable multimodal RBC signatures integrating morphology, deformability, hydration, ion transport, metabolism, redox status, molecular profiles, adhesion, and extracellular-vesicle characteristics. Successful implementation will depend on shared datasets, interpretable analytical models, harmonized reference ranges, and prospective multicenter validation. The most pressing research priority is to determine whether standardized RBC functional measurements improve clinical decisions or patient outcomes beyond established diagnostic pathways.

## 8. Conclusions

Red blood cells are active participants in vascular and systemic homeostasis. Their mechanical, metabolic, redox, and signaling properties influence microvascular perfusion and vascular function while providing clinically relevant information beyond the conventional blood count. Altered RBC phenotypes may identify primary red-cell disorders or record the cumulative effects of systemic metabolic, inflammatory, renal, cardiovascular, infectious, and malignant disease.

These same biological properties create therapeutic opportunities. RBC-directed treatments can improve hydration, metabolism, hemoglobin function, survival, and pathological vascular interactions, whereas intact RBCs and RBC-derived materials offer adaptable drug-delivery platforms. Translation into precision medicine will depend on selecting clinically meaningful combinations of functional and molecular measurements, standardizing their application, and validating their relationship with patient outcomes. Prospective multicenter studies must first establish which standardized RBC functional measurements change clinical decisions or patient outcomes beyond established investigations.

## Figures and Tables

**Table 3:** Principal therapeutic targets in red blood cells.

Therapeutic Domain	Principal Targets or Strategies	Expected Effect	Main Limitations and Representative References
Membrane and hydration	Modulation of the PIEZO1-Ca^2+^-KCNN4/Gardos pathway; membrane stabilization; antioxidant protection	Prevention of pathological dehydration, reduced cytoplasmic viscosity, and preserved deformability	Systemic PIEZO1 inhibition may cause off-target effects; improved rheology may not prevent clinical complications [6,10,43]
Hemoglobin function	HbF induction; inhibition of HbS polymerization; oxygen-affinity modulation; reduction of hemoglobin oxidation	Reduced sickling and oxidative membrane injury; improved microvascular transit	Higher oxygen affinity may impair tissue oxygen unloading; responses vary among therapies and patients [44,45,46,49,50,51,52]
Metabolism and survival	Pyruvate kinase activation; restoration of ATP; enhancement of antioxidant defense	Improved membrane homeostasis, reduced hemolysis, and prolonged RBC survival	Response depends on genotype and residual enzyme activity; effects on 2,3-BPG require consideration [5,47]
Pathological RBC interactions	Inhibition of endothelial adhesion and inflammation; complement blockade; scavenging of cell-free hemoglobin and heme	Reduced vaso-occlusion, thrombosis, intravascular hemolysis, and vascular toxicity	Redundant pathways, infection risk, cost, and limited clinical evidence for heme-scavenging therapies [18,48,49,53]

The four therapeutic domains are interconnected. Improvement in an isolated laboratory parameter does not necessarily predict clinical benefit; assessment should integrate RBC hydration, deformability, hemolysis, oxygen delivery, survival, and vascular outcomes. Abbreviations: 2,3-BPG, 2,3-bisphosphoglycerate; ATP, adenosine triphosphate; Ca^2+^, calcium; HbF, fetal hemoglobin; HbS, sickle hemoglobin; KCNN4, potassium calcium-activated channel subfamily N member 4; PIEZO1, piezo-type mechanosensitive ion channel component 1; RBC, red blood cell.

**Table 4:** Comparison of red blood cell-based therapeutic platforms.

RBC-Based Platform	Construction or Cargo	Principal Advantages	Main Limitations	Development Stage and Representative References
Intact RBC carrier	Intracellular encapsulation or surface attachment of enzymes, small molecules, biologics, antibodies, or nanoparticles	High loading capacity; cargo protection; prolonged release; access to circulating or vascular targets	Loading may impair deformability, membrane integrity, hemocompatibility, or circulatory survival; individualized manufacturing	Clinical and translational development [11,54,61]
RBC membrane-coated nanoparticle	Synthetic drug- or imaging-agent core enclosed by an RBC-derived membrane shell	Biomimetic immune camouflage; prolonged circulation; adaptable core and targeting	Membrane-source variability; incomplete coating; altered protein orientation; scale-up, sterility, and storage challenges	Predominantly preclinical [55,56]
RBC-derived extracellular vesicle	Natural or engineered vesicles carrying proteins, RNA, small molecules, or therapeutic compounds	Cell-free delivery; low nuclear-genetic-material risk; potential functionalization and targeting	Potential coagulation or inflammatory effects; residual hemoglobin; batch variability; uncertain potency assays	Preclinical and early translational development [26,57,58]
Engineered erythroid cell	Ex vivo modified erythroid precursors producing enucleated cells that express or contain therapeutic proteins	Programmable expression; prolonged circulation; enucleated final product; potential personalized therapy	Expansion and enucleation efficiency; genetic stability; manufacturing cost; complex regulation	Preclinical and early clinical development [59,60]

Development stage describes the overall maturity of each platform and may differ between individual products. All approaches require assessment of loading efficiency, membrane integrity, deformability, hemocompatibility, cargo stability, biodistribution, circulatory survival, and manufacturing reproducibility. Evidence base distinguishes human studies from cell, animal or conceptual studies; a platform-level classification does not confer approval on any product. Figure 4 correspondence: blue, intact RBC carrier; teal, RBC membrane-coated nanoparticle, with a teal therapeutic core enclosed by a red dashed RBC-membrane ring; violet, RBC-derived extracellular vesicle; and green, engineered erythroid cell. Solid navy arrows indicate that all four platforms converge on the common objectives of prolonged circulation and targeted therapeutic delivery. Abbreviations: RBC, red blood cell; RNA, ribonucleic acid.

**Table 5:** Proposed integrated red blood cell functional profile.

Domain	Core Measurements	Clinical Contribution and Representative References
Morphology	Digital blood-film analysis; automated cell-shape classification; CBC and reticulocyte indices; automated image segmentation	Detection and quantification of abnormal RBC populations [34,39]; routine baseline screening
Mechanics	Ektacytometry; microfluidic transit and occlusion; oxygen-gradient ektacytometry; single-cell analysis	Assessment of deformability and microcirculatory performance [22,27,28,29,30,31]; hypoxic sickling, patient stratification, and treatment response
Hydration and ion transport	Osmoscan parameters; intracellular cations; PIEZO1/KCNN4-related transport	Recognition of dehydration, overhydration, and permeability disorders [6,10,27,28]; monitoring of ion-channel modulators
Metabolism	ATP; 2,3-BPG; glycolytic intermediates; enzyme activity; hemolysis markers	Assessment of energetic capacity and metabolic dysfunction [5,9,20,36]; monitoring of metabolic therapy
Redox status	Glutathione; hemoglobin oxidation; lipid peroxidation	Evaluation of antioxidant reserve and oxidative membrane injury [8,20]
Molecular profile	Genomics; proteomics; metabolomics; lipidomics; ion-transport analysis; integrated multimodal decision-support models (exploratory)	Identification of causal pathways and interpretation of genetic variants [28,36,37,42]; patient stratification
Cellular communication	Endothelial adhesion; extracellular-vesicle analysis	Assessment of vascular, inflammatory, and procoagulant interactions [12,13,14,18,26]; monitoring of vascular interventions

The domains should be interpreted collectively and in relation to the clinical phenotype. Longitudinal assessment may support diagnosis, patient stratification, interpretation of genetic variants, and therapeutic monitoring. Harmonized preanalytical and analytical procedures are required for clinical implementation. Prioritization is a proposed pathway, not a validated clinical algorithm; assay availability and clinical utility differ between conditions. Automated image analysis and machine-learning-assisted microfluidic phenotyping have been demonstrated in curated research datasets, whereas multimodal decision-support integration remains exploratory and requires prospective multicenter validation. Clinical adoption also requires calibrated instruments, reference ranges, interoperable data, interpretable models, and evaluation of cost-effectiveness, throughput, turnaround time, and a tiered testing strategy. Abbreviations: 2,3-BPG, 2,3-bisphosphoglycerate; ATP, adenosine triphosphate; KCNN4, potassium calcium-activated channel subfamily N member 4; PIEZO1, piezo-type mechanosensitive ion channel component 1; RBC, red blood cell.

## Data Availability

No new data were created or analyzed in this study. Data sharing is not applicable to this article.
